# Mode of Detecting Morbid Bile

**Published:** 1846-04

**Authors:** 


3. *Mode of detecting Morbid Bile.*—In some diseases, however, as in cholera, &c., Dr. Heller has found that the colouring matter of bile may undergo a very considerable morbid change, in consequence of which, when treated with nitric acid, it assumes at once a red instead of a green colour. He has found that for bile which has undergone this change ammonia is a better and a more certain test than nitric acid, for although this latter reagent will detect it when in any abundance, yet it is apt to prove deceptive in cases where urine is the fluid undergoing examination, especially if much hæmatosine be present, which substance becomes more or less red by nitric acid. In using the ammonia test, a small quantity only should be at first dropped in, immediately upon doing which a bright red colour is struck; more of the ammonia may then be added until a reddish-brown fluid is obtained. In this way it is possible to detect the presence of a very minute quantity of altered bile pigment, even when nitric acid fails to afford the smallest evidence of its existence.—*Ibid.*

